# Effects of* Achyrocline satureioides* Inflorescence Extracts against Pathogenic Intestinal Bacteria: Chemical Characterization, In Vitro Tests, and In Vivo Evaluation

**DOI:** 10.1155/2017/4874865

**Published:** 2017-09-07

**Authors:** Karla Suzana Moresco, Alexandre Kleber Silveira, Fares Zeidán-Chuliá, Ana Paula Folmer Correa, Rafael R. Oliveria, Adriana Giongo Borges, Lucas Grun, Florencia Barbé-Tuana, Ariane Zmozinski, Adriano Brandelli, Maria Goretti Rodrigues Vale, Daniel Pens Gelain, Valquiria Linck Bassani, José Cláudio Fonseca Moreira

**Affiliations:** ^1^Departamento de Bioquímica, Universidade Federal do Rio Grande do Sul (UFRGS), Porto Alegre, RS, Brazil; ^2^Faculdade de Ciências da Saúde, Centro Universitário Ritter dos Reis (UniRitter), Porto Alegre, RS, Brazil; ^3^Instituto de Ciência e Tecnologia de Alimentos, Universidade Federal do Rio Grande do Sul (UFRGS), Porto Alegre, RS, Brazil; ^4^Instituto de Tecnologia do Petróleo, Pontíficia Universidade Católica do Rio Grande do Sul (PUCRS), Porto Alegre, RS, Brazil; ^5^Instituto de Quimica, Universidade Federal do Rio Grande do Sul (UFRGS), Porto Alegre, RS, Brazil; ^6^Faculdade de Farmácia, Universidade Federal do Rio Grande do Sul (UFRGS), Porto Alegre, RS, Brazil

## Abstract

Three* Achyrocline satureioides* (AS) inflorescences extracts were characterized: (i) a freeze-dried extract prepared from the aqueous extractive solution and (ii) a freeze-dried and (iii) a spray-dried extract prepared from hydroethanol extractive solution (80% ethanol). The chemical profile, antioxidant potential, and antimicrobial activity against intestinal pathogenic bacteria of AS extracts were evaluated. In vitro antioxidant activity was determined by the total reactive antioxidant potential (TRAP) assay. In vivo analysis and characterization of intestinal microbiota were performed in male Wistar rats (saline versus treated animals with AS dried extracts) by high-throughput sequencing analysis: metabarcoding. Antimicrobial activity was tested in vitro by the disc diffusion tests. Moisture content of the extracts ranged from 10 to 15% and 5.7 to 17 mg kg^−1^ of fluorine. AS exhibited antioxidant activity, especially in its freeze-dried form which also exhibited a wide spectrum of antimicrobial activity against intestinal pathogenic bacteria greater than those observed by the antibiotic, amoxicillin, when tested against* Bacillus cereus* and* Staphylococcus aureus*. Antioxidant and antimicrobial activities of AS extracts seemed to be positively correlated with the present amount of flavonoids. These findings suggest a potential use of AS as a coadjuvant agent for treating bacterial-induced intestinal diseases with high rates of antibiotic resistance.

## 1. Introduction

Many bacterial pathogens associated with epidemics of gastrointestinal tract disorders in humans, such as* Escherichia coli*,* Salmonella* spp., and* Staphylococcus aureus*, have evolved multidrug-resistant forms subsequent to antibiotic use [1]. The extensive use of antibiotics over the last decade has led to the emergence of bacterial resistance and the dissemination of resistant genes among pathogenic microorganisms [2]. In recent years, several associations between common chronic human disorders and an altered composition and function of the gut microbiome have been reported [3–6]. Numerous studies have shown that changes in some bacterial phyla of the intestinal microbiota [3, 5, 7], such as Proteobacteria and Bacteroidetes/Firmicutes, can be considered indicators of dysbiosis.

Diseases of the gastrointestinal tract are induced by oxidative stress and overproduction of reactive oxygen species (ROS), which accumulate under abnormal conditions and contribute to the rapid development of inflammation [8]. Many of the chemical constituents of plants, such as flavonoids, have been described as scavengers of the superoxide anion, hydroxyl radicals, and peroxyl radicals, as well as being inhibitors of key enzymes of mitochondrial respiration [9–11].

The use of plant extracts in folk medicine has been widely proposed as an important factor for the positive selection of intestinal organisms that metabolize or resist these secondary plant compounds. Many dietary polyphenols are not absorbed in the small intestine and can interact with colonic microbiota, producing diverse metabolites with a variety of physiological roles [12]. These phenomena may modulate gut microbiota and, consequently, alter the Bacteroidetes/Firmicutes balance [12, 13].

Infusions from* Achyrocline satureioides* (Asteraceae) or “Marcela” inflorescences are frequently used to treat several human diseases, many of them related to gastrointestinal dysfunction [14]. Previous studies of the composition of* A. satureioides* have demonstrated the presence of flavonoids, which have been shown to inhibit lipid peroxidation by scavenging ROS and chelating metal ions responsible for ROS generation [15]. Other phenolic constituents, including achyrofuran and phloroglucinol derivatives, have shown some antibacterial effects [16, 17] without causing any bacterial resistance or adverse side effects. However, to the best of our knowledge, no reports regarding the relationship between aglycone and achyrobichalcone, the major flavonoids present in* A. satureioides* inflorescences, and the intestinal activity of pathogenic bacteria have been reported.

In this context, the aim of this study was to investigate the chemical profile, the antioxidant potential in vitro, and the antimicrobial activity of* A. satureioides* inflorescence extracts against intestinal pathogenic bacteria (both gram-positive and gram-negative) as well as characterize alterations in the intestinal microbiota of rats treated with* A. satureioides* extracts.

## 2. Material and Methods

### 2.1. Plant Material

*A. satureioides* inflorescences were purchased from Centro de Pesquisas Químicas, Biológicas e Agronômicas (CPQBA, Universidade de Campinas, Brazil). The plant samples were collected and dried (room temperature) in May 2013 and identified as Cultivar CPQBA/2; they were registered at Ministério Agricultura, Pecuária e Abastecimento (MAPA-Brazil), as number 22975.

### 2.2. Chemicals

The following compounds were used for the experiments and were of high performance liquid chromatography (HPLC) grade: methanol (JT Baker, USA), acetonitrile (Tedia, Brazil), and phosphoric acid (Merck). Catalase (CAT, EC 1.11.1.6); superoxide dismutase (SOD, EC 1.15.1.1); thiobarbituric acid (TBA); hydrogen peroxide (H_2_O_2_); and the standards, quercetin, and luteolin were purchased from Sigma-Aldrich (St. Louis, MO, USA). The flavonoid 3-O-methylquercetin was purchased from Extrasynthese (France).

### 2.3. Preparation of* A. satureioides* Extracts

Three extracts from* A. satureioides* inflorescences were prepared, including an aqueous extract, freeze-dried extract, and spray-dried extract. The plant : solvent ratio was 7.5 : 100 (w/v) for the three extracts. The aqueous extractive solution was prepared via decoction and then freeze-dried. The freeze-dried and spray-dried extracts were obtained by maceration of inflorescences in 80% ethanol (v/v). The extraction time was eight days, with occasional stirring [18]. The resulting extractive solution was filtered, and the supernatant was freeze-dried (frozen at −80°C and subsequently dried in a freeze-dryer (Edwards Modulyo 4K, Irvine, USA) at −60°C and pressure of −10^−2^ bar) or spray-dried (Spray Dryer Buchi B-290, with a two-component nozzle and current flow, under the following operating conditions: inlet temperature, 160 + 2°C; output temperature 140 + 2°C; feed rate, 3 mL/min; and spraying pressure, 2 bar [19]). The spray-dried extract contained 50% extractives, 33.4% colloidal silicon dioxide, and 16.6% polysorbate 80.

### 2.4. Bromatological Analysis

All chemical composition analyses of* A. satureioides* inflorescences and the corresponding extracts were performed in triplicate, according to previously described methods [20]. The protein content was quantified using the Kjeldahl method, with a converting factor of 5.75. Lipid analyses were performed by extraction with ethyl ether using a Soxhlet extractor. Reducing, nonreducing, and total sugar analyses were carried out according to the Lane-Eynon method [20]. The loss on drying analysis was conducted per the method outlined by the Association of Official Agricultural Chemists [20].

### 2.5. Elemental Content

The determination of cadmium, lead, chromium, and fluorine content was carried out using a contrAA® 700 model high-resolution continuum source graphite furnace atomic absorption spectrometer (HR-CS SS-GF AAS, Analytic Jena AG, Germany). The* A. satureioides* and extract powder source samples were ground in an A-11 basic micromill (IKA-Werke, Germany) and sieved through a 150 *μ*m polyester mesh to improve particle size distribution. Larger particles that did not pass through the mesh were ground and sieved again until the entire sample passed through. The samples were then dried at 50°C for 3 h in an oven to eliminate absorbed moisture. The dried samples were stored in sealed plastic vials until further processing. The samples (0.01 to 0.30 mg) were weighed directly on the direct solid sampling (SS) platform and introduced into the HR-CS SS-GF AAS to determine cadmium, chromium, and lead concentrations. The instrumental parameters were optimized, and the method was adopted per a previous report [21].

To determine fluorine content, samples (0.01 to 0.15 mg) were weighed directly onto the SS platform and introduced into the HR-CS SS-GF MAS. Then, 10 *μ*L of a solution containing 1500 mg L^−1^ Ca (15 *μ*g Ca) was injected directly onto the sample to form CaF diatomic molecules. The instrumental parameters were optimized, and the method was adopted per a previous report [22].

### 2.6. Flavonoid Content

Approximately 20 mg of each extract was dissolved in a liquid chromatography (LC) system (methanol : 16 mM phosphoric acid (50 : 50, v/v)) and transferred to a 100 mL volumetric flask. The solution was filtered through a 0.45 *μ*m membrane filter (Millipore-HVHP, MA, USA), and the supernatant was injected into the LC equipment. Evaluations for each sample were repeated three times. LC analysis of* A. satureioides* extracts was performed following the method described by Bidone et al. [18]. Briefly, the LC equipment comprised a Shimadzu LC-10A system equipped with an LC-10 AD pump and a CBM- 10A system controller. The system was maintained at 30 + 1°C, and the programmed injection volume was 20 *μ*L. Samples were diluted in methanol : 16 mM phosphoric acid (50 : 50, v/v). The method specificity was determined with a Shimadzu LC-20A system equipped with an LC-20 AT pump, a CBM- 20A system controller, an SIL-20A autosampler, and an SPD-M20A diode array detector.

### 2.7. Total Reactive Antioxidant Potential

Total reactive antioxidant potential (TRAP) was used as an index of the nonenzymatic antioxidant capacity of* A. satureioides* extracts. Briefly, this assay is based on the ability of samples to quench peroxyl radicals generated by 2,2′-azo-bis(2-amidinopropane) dihydrochloride (AAPH). The reaction contained AAPH (10 mM) and luminol (35 *μ*M) dissolved in 0.1 M glycine buffer (pH 8.6). After stabilizing for 2 h at room temperature, different concentrations of* A. satureioides* extract were added to the system to determine their antioxidant potential. Results are expressed as the area under the curve (AUC), and the total antioxidant properties (TAR) of each* A. satureioides* extract were compared to the system. A smaller AUC in relation to the system indicates a higher total reactive antioxidant potential. TAR is closely related to the quality of the antioxidants within the sample. In our study, TAR was calculated as the ratio of chemiluminescence in the absence of sample (*I*_*o*_)/chemiluminescence immediately after sample addition (*I*). Higher TAR values represent a greater antioxidant capacity of the sample [23].

### 2.8. Ferric Ion Reducing Antioxidant Power (FRAP) Assay

This assay determines the ability of antioxidants to reduce iron. FRAP activity was measured according to the method of Benzie and Strain [24], with modifications. Briefly, we mixed 90 mL diluted extract (20 mg/mL) with 270 mL distilled water and 2.7 mL FRAP reagent [2.5 mL 10 mM 2,4,6-Tris(2-pyridyl)-s-triazine (TPTZ), 2.5 mL 20 mM ferric chloride, and 25 mL 0.3 M acetate buffer, pH 3.6] in a dark environment. The mixture was vortexed and incubated at 37°C for 30 min; absorbance at 595 nm was then recorded. A standard curve using a 2 mM ferrous sulfate (Fe^+2^ source) standard was created to calculate the amount of Fe^+2^ produced during the extract-induced reduction of Fe^+3^. Equivalent concentration was used as a parameter to define the concentration of antioxidant having a ferric TPTZ-reducing ability equivalent to that of 1 mM FeSO_4_·7H_2_O.

### 2.9. Fe^2+^ Chelating Activity Assay

The ferrous ion-chelating ability of the extracts was determined as previously described [25]. The reaction mixture, containing 200 mL diluted extract (20 mg/mL), 25 mL FeSO_4_ (2 mM), and 100 mL FerroZine™ (5 mM), was shaken well and incubated for 10 min at room temperature. Ethanol (1.675 mL) was then added to the mixture to stop the reaction. Ethylenediaminetetraacetic acid (EDTA), a known chelating agent, was used to construct a standard curve (0.5–10 *μ*g/mL final concentration). Absorbance of the FerroZine-Fe^+2^ complexes was measured at 562 nm, and the results are expressed as mg EDTA equivalents/g dry extract weight.

### 2.10. Hydroxyl Radical Scavenging

This assay measures the antioxidant activity of a substance against hydroxyl radicals. The formation of hydroxyl radicals from the Fenton reaction is used to quantify the oxidative degradation of 2-deoxyribose (2-DR). 2-DR is incubated with a system that generates hydroxyl radical and degrades into malondialdehyde (MDA), which condenses with thiobarbituric acid (TBA) to form a pink chromophore. The chromophore was quantified spectrophotometrically at a wavelength of 532 nm, as previously described [26].

### 2.11. Thiobarbituric Acid (TBARS) Assay

The TBARS assay indirectly measures lipid peroxidation in biological systems. The assay uses TBA to react with lipoperoxides such as MDA. The reaction of MDA with TBA produces a pink-red color, which is detected by absorbance at 532 nm [27].

### 2.12. Characterization of Intestinal Microbiota

Male Wistar rats (20 animals) were randomly divided into two groups (ten rats per group) that received saline (1 mL kg^−1^) or* A. satureioides* extract (35 mg kg^−1^, equivalent to the consumption of 150 mL tea per day). Rat feces were collected on the counter for handling animals in the vivarium at the Universidade Federal do Rio Grande do Sul after cleaning with 70% alcohol on day 0 (D0) and D21 after treatment. The feces were placed in wells that had been presterilized for 20 min under UV light from a laminar flow cabinet. To better represent the diversity of each group, the feces were pooled where 4 wells were made for each per group on the tubes. All experiments were approved by the Institutional Animal Care and Use Committee at the Federal University of Rio Grande do Sul (IACUC #25449).

### 2.13. DNA Extraction and Amplification

Total DNA was extracted immediately after collection using the QIAmp DNA Stool Mini Kit (QIAGEN, Hilden, Germany), following the manufacturer's instructions. After extraction, DNA was stored at −20°C. Partial 16S rRNA gene fragments were amplified using universal primers 515F and 806R [28]. The PCR conditions included one initial denaturation step at 94°C for 45 s, 30 cycles of denaturation for 45 s at 94°C, annealing for 45 s at 50°C, and extension for 1 min at 72°C, with one final extension step for 7 min at 72°C. After amplification, 16S rRNA bands were selected and purified from a 3% agarose gel using the Wizard® SV Gel and PCR Clean-Up System kit (Promega, Madison, WI, USA).

### 2.14. High-Throughput Sequencing Analysis, Metabarcoding

The 16S rRNA reads were generated via high-throughput sequencing and were submitted for quality control to retain sequences with a minimum length of 100 bp and trim sequences to remove low quality bases (minimum Phred score of 30) using PRINSEQ [29]. The remaining sequences were dereplicated, sorted by decreasing read abundance, and then filtered to exclude singletons using USEARCH v7.0.1090 [30]. Clusters were assembled using a minimum identity of 99%, and chimeras were removed using the Ribosomal Database Project (RDP) reference database [31]. The taxonomic assignment was obtained using Quantitative Insights into Microbial Ecology (QIIME v1.7) [32]. Sequences were clustered and assigned to operational taxonomic units (OTUs), which were selected based on 97% sequence similarity. Taxonomic data were achieved through the classification algorithm, using the 97% OTUs version of GreenGenes 13.8 [33].

### 2.15. Determination of Antibacterial Activity

The antibacterial activity of* A. satureioides* extracts against the bacteria* Listeria monocytogenes* (ATCC 15131),* B. cereus* (ATCC 9634),* S. aureus* (ATCC 1901),* Salmonella enteritidis* (ATCC 13076),* Listeria innocua*,* Pseudomonas*,* Aeromonas* (ATCC 27853),* Escherichia coli* (ATCC 8739), and* Corynebacterium fimi* (NCTC 7547) was determined according to the procedure of Motta and Brandelli [34], with modifications. Indicator microorganisms, at a concentration of 10^8^ CFU mL^−1^ in saline solution (0.85% NaCl, w/v), were inoculated with a swab onto brain heart infusion (BHI) agar plates.

A pilot trial was initially performed with three extracts to evaluate the bacterial growth inhibiting capacity of the extracts. Aliquots of 20 *μ*L freeze-dried extract (50, 100, and 200 mg/mL) were spotted on the freshly prepared indicator strain lawn, and plates were incubated at the optimal temperature for each test microorganism. Subsequently, zones of growth inhibition (represented by clear haloes) were measured and depicted as an inhibition zone (mm).

Based on the results of this initial experiment, antibacterial and bacteriolytic activities were observed with the freeze-dried hydroalcoholic extract only. Inhibitory capacity was the greatest against* B. cereus* (ATCC 9634),* S. aureus* (ATCC 1901), and* S. enteritidis* (ATCC13076). The reference antibiotics used as positive controls were chloramphenicol, amoxicillin, and ciprofloxacin.

Bacterial cells were cultivated in casein soy broth (17 g enzymatic digest of casein, 3 g enzymatic digest of soybean meal, 2.5 g dextrose, 5 g sodium chloride, and 2.5 g dipotassium phosphate per liter, pH 7.3) at 27°C. The overnight bacterial cultures were harvested by centrifugation (10 min, 3000 rpm) and resuspended in fresh casein soy broth. Bacterial concentrations were adjusted to approximately 5 × 10^7^ cells/mL (OD600 ~0.2). Broth bacterial cultures and test samples were mixed for optical density measurements.

### 2.16. Statistical Analysis

Data obtained from animal experiments are expressed as means ± standard error of the mean. Statistical differences between the treatments and the controls were assessed by Student's *t*-test and Bonferroni. A value of *P* < 0.05 was considered statistically significant.

## 3. Results

### 3.1. Bromatological Analysis and Composition of* A. satureioides* Extracts

**Figure 1:**
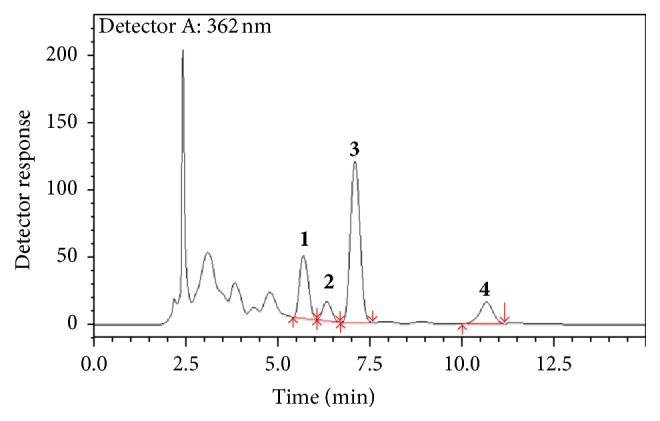
Chromatographic profile (Rp-HPLC) of* Achyrocline satureioides* freeze-dried extracts prepared from a hydroethanol extractive solution. (**1**) Luteolin, (**2**) quercetin, (**3**) 3-o-methylquercetin, and (**4**) achyrobichalcone are shown. Absorbance was detected at 362 nm.

Our results show that the level of reducing sugars and lipids in the aqueous extract was lower than that in the source plant material (Table 1). Loss on drying measurements of the extracts ranged from 5.7 to 17%, with the spray-dried extract having the lowest values. Analyses of heavy metals (lead, cadmium, and chromium) and fluorine content in the extracts and original plant material revealed that the aqueous extract contained higher levels of lead, cadmium, and fluorine than the plant material and the two extracts prepared from the hydroethanol extractive solution. The plant material and all extracts contained concentrations of heavy metals that were below harmful limits (Table 2). Finally, three major flavonoid aglycones and a chalcone were quantified, namely, quercetin, 3-*O*-methylquercetin, luteolin, and achyrobichalcone, respectively (Figure 1). Total flavonoid content of the freeze-dried extract (132 mg/g) was slightly higher than that found in the spray-dried extract (129.7 mg/g). The flavonoid content in both the freeze-dried and spray-dried extracts was approximately twofold higher than that found in the freeze-dried aqueous extract (54.23 mg/g) (Table 3). These results demonstrate that 80% ethanol (v/v) is more efficient for extracting the three flavonoid aglycones and chalcone from the inflorescences than water alone. Further, the two drying methods did not influence the content of the extracts.

### 3.2. Antioxidant Properties of* A. satureioides* Extracts

**Figure 2:**
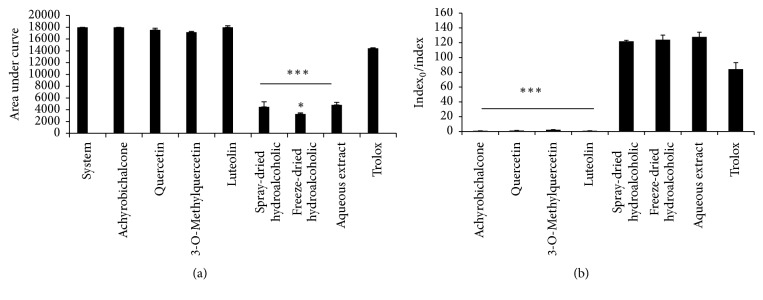
(a) In vitro effects of* Achyrocline satureioides* extracts on total radical-trapping antioxidant potential (TRAP). Data are presented as means  ±  SEM of three experiments. Black bars represent* A. satureioides* extracts and flavonoids compared to the system (i.e., solvents without sample and Trolox = control). ^*∗∗∗*^*P* < 0.0001 extracts versus system (Student's *t*-test) and ^*∗*^*P* < 0.05 freeze-dried extract versus aqueous extract and spray-dried extract. (b) Total antioxidant reactivity (TAR) index, which is related to antioxidant quality, is shown. ^*∗∗∗*^*P* < 0.0001 flavonoids versus Trolox. Aqueous extract: freeze-dried extract prepared from* A. satureioides* decoction. Both freeze-dried and spray-dried extracts were prepared from an* A. satureioides* hydroethanol extractive solution (80% ethanol). All extractive solutions were prepared using a plant : solvent ratio of 7.5% (w/v).

**Figure 3:**
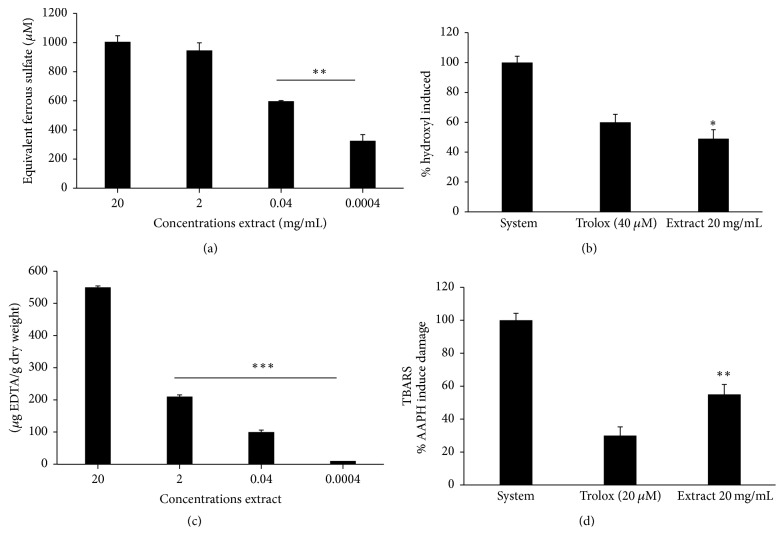
(a) Ferric ion reducing antioxidant power (FRAP* Assay*). Low concentrations (0.0004 and 0.004 mg/mL) of* Achyrocline satureioides* freeze-dried extract versus high concentrations (2 and 20 mg/mL) of* A. satureioides* freeze-dried extract are shown (^*∗∗*^*P* < 0.005). (b) Ferrozine assay. Results from the freeze-dried extract versus system (^*∗*^*P* < 0.05) are shown. Hydroxyl radical scavenging properties of (c) low* A. satureioides* extract concentrations (0.0004, 0.004, and 2 *μ*g ethylenediaminetetraacetic acid (EDTA)/g dry weight) versus high concentrations (20 EDTA/g dry weight) (^*∗∗∗*^*P* < 0.005) are shown. (d) Thiobarbituric acid assay. Results from the freeze-dried extract versus system are shown (^*∗∗*^*P* < 0.005).

The dried* A. satureioides* extracts all exhibited antioxidant activity, especially the freeze-dried form. At a concentration of 34 *μ*g/mL, luminescence counts (AUC) were lower for the dried extracts than for the system (*P* < 0.0001) (Figure 2). The antioxidant potential was higher for the freeze-dried extract, which showed a better quenching potential against peroxyl radical than that of the spray-dried and aqueous extracts, as determined by the TRAP assay (*P* < 0.01 and *P* < 0.01, resp.) (Figure 2(a)). When the flavonoid and chalcone content were analyzed in the extracts separately, we observed that the antioxidant activity could be attributed to synergism between the four extract compounds rather than a specific phenolic constituent. Lower AUC values represent higher antioxidant potential in the TRAP assay, whereas TAR values are directly proportional to the antioxidant properties (Figure 2(b)). Reduction equivalents of the freeze-dried extract (0.0004 mg) were equal to 325 *μ*M ferrous ions, as determined by FRAP analyses (Figure 3(a)). In addition, the hydroxyl radical scavenging activity of the extracts was similar to that of the positive control (6-hydroxy-2,5,7,8-tetramethylchroman-2-carboxylic acid [Trolox]) (Figure 3(b)). Further, the extracts possessed significant ferrous ion-chelating properties, as determined via the FerroZine assay (Figure 3(c)). Finally, the extracts were efficient for protecting against lipid peroxidation, presenting values close to those exerted by Trolox and being slightly above those of the negative control (Figure 3(d)).

### 3.3. In Vivo Analysis of Intestinal Microbiota in Rats Supplemented with* A. satureioides* Extracts via High-Throughput Sequencing Analysis, Metabarcoding

**Figure 4:**
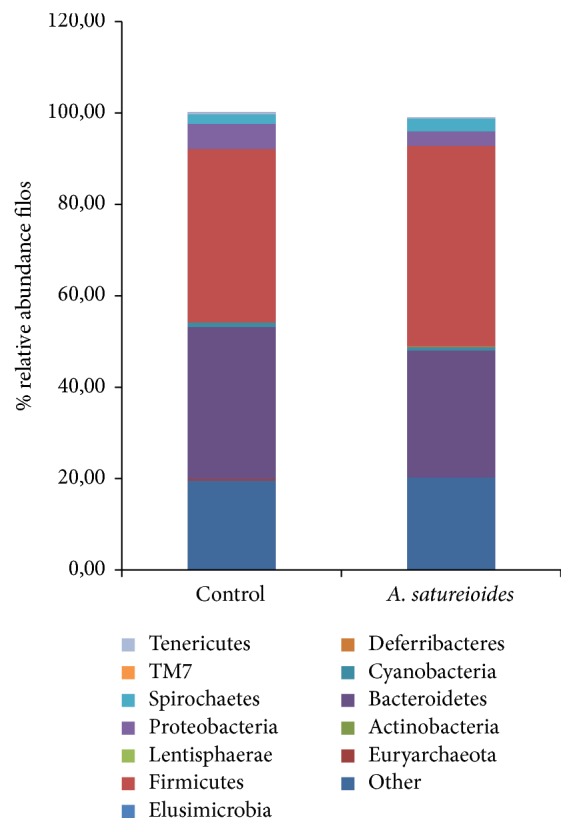
Profile of the intestinal microbiota of rats supplemented with saline (control) and freeze-dried extract prepared from an* Achyrocline satureioides* hydroethanol extractive solution (80% ethanol). The specific filos of the figure are Proteobacteria, Bacteroidetes, Firmicutes, Cyanobacteria, Deferribacteres, Spirochaetes, and Other.

**Figure 5:**
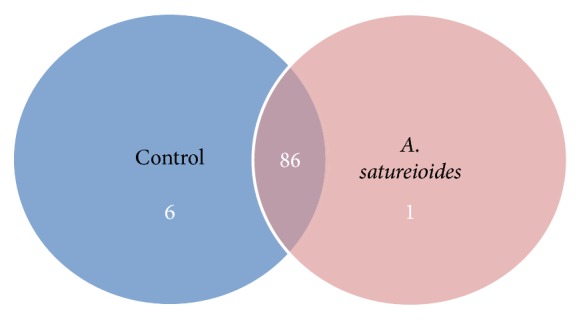
Venn diagram showing genotypes found in the freeze-dried extract group prepared from an* Achyrocline satureioides* hydroethanol extractive solution (80% ethanol) versus control group.

We used high-throughput sequencing to obtain a total of 3, 217, and 215 reads, which were grouped into OTUs, with a 97% similarity cut-off level at different taxonomic levels (i.e., phylum, class, order, family, and genus). The primary gut microbiota of the rats comprised Euryarchaeota, Actinobacteria, Bacteroidetes, Cyanobacteria, Deferribacteres, Elusimicrobia, Firmicutes, Lentisphaerae Proteobacteria, Spirochaetes, Tenericutes, and TM7 (Figure 4). The abundance and diversity of the gut microbiota in rats supplemented with* A. satureioides* freeze-dried extract were not significantly different from those of the control. The proportion of bacterial phyla present in the intestinal microbiota of rats that received the freeze-dried extract was not significantly different from that of the controls, as shown in the Venn diagram displaying genotypes of the control group versus the group that received treatment (Figure 5). Bacteria from the Proteus genus were exclusively found in the group treated with* A. satureioides*.

### 3.4. In Vitro Testing of* A. satureioides* Antibacterial Properties against Intestinal Pathogens

**Figure 6:**
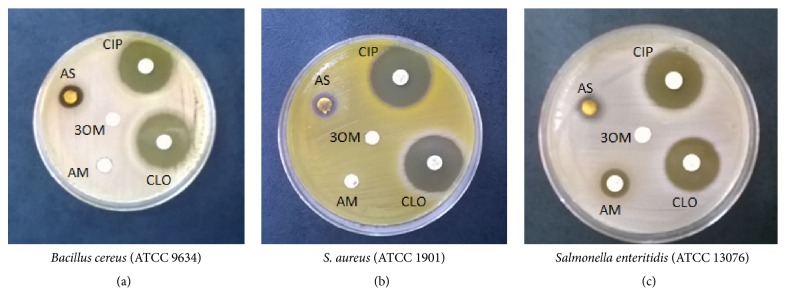
Plate inhibition test of the* Achyrocline satureioides* (AS) freeze-dried extract (200 mg/mL), prepared from an* A. satureioides* hydroethanol extractive solution (80% ethanol), and the major flavonoid-aglycone 3-O-methylquercetin (3OM) against (a)* Bacillus cereus* (ATCC 9634), (b)* Staphylococcus aureus* (ATCC 1901), and (c)* Salmonella enteritidis* (ATCC13076). Antibiotic references used as positive controls include chloramphenicol (CLO) (30 *μ*g), amoxicillin (AM) (30 *μ*g), and ciprofloxacin (CIP) (5 *μ*g).

A pilot trial was conducted using the three extracts to determine the growth inhibiting capacity for different bacteria. The freeze-dried extract prepared from the 80% ethanol (v/v) extractive solution exhibited a broad spectrum of antimicrobial activity (100–200 mg/mL). Among all the bacteria tested, we chose the three strains showing the greatest inhibition for further testing. Specifically, the antibacterial effects of freeze-dried* A. satureioides* extracts were compared to those of chloramphenicol, amoxicillin, and ciprofloxacin (antibiotics), as well as 3-O-methylquercetin (the primary flavonoid in the* A. satureioides* extracts) in strains of* B. cereus* (ATCC 9634),* Staphylococcus aureus* (ATCC 1901), and* S. enteritidis* (ATCC 13076) (Table 4). As can be seen in (Figure 6), gram-positive strains are intrinsically resistant to amoxicillin, and evidence indicates that the antibacterial effects of* A. satureioides* extracts were higher than those exerted by amoxicillin (used as a positive control) when tested against* B. cereus* and* S. aureus*. However, additional tests are required for further confirmation.

## 4. Discussion

Currently, resistance to antimicrobials is a global problem of increasing importance that endangers the efficacy of antibiotics, which have transformed medicine and saved millions of lives [35]. Treatments for resistant infections cost the US health care system an estimated $21 billion to $34 billion annually [36]. In the present study, we characterized the chemical composition, antioxidant properties, and antimicrobial activities exerted by dried* A. satureioides* inflorescence extracts prepared from aqueous or hydroethanol extractive solutions. We selected the freeze-dried extract, obtained from an 80% ethanol (v/v) extractive solution, to perform both in vivo and antimicrobial activity experiments; this extract had the highest flavonoid content and significantly higher antioxidant activity than the other extracts. Furthermore, the freeze-dried extract exhibited a wide spectrum of antimicrobial activity (100 to 200 mg/mL) against intestinal pathogenic bacteria. We observed that the antibacterial effects of the freeze-dried extract were greater than those exerted by amoxicillin (an antibiotic used as a positive control) when tested against* B. cereus* and* S. aureus*. One hypothesis is that such effects are attributable to the lipophilic compounds in the extract interacting with the hydrophobic part of the bacterial membrane, thereby affecting membrane anisotropy and dipolar organization.

Screening natural products for antimicrobial activity has resulted in the discovery of higher plants as a potential source of new antibacterial agents [37], and the use of natural products derived from plants is a potential therapeutic alternative to antibiotics. Further, screening chemical compounds coming from natural products for antimicrobial activity represents an alternative strategy for the development of novel drugs. For centuries, preparations containing flavonoids as the principal physiologically active constituent have been used to treat human diseases [38]. Some researchers have reported synergy between naturally occurring flavonoids and other antibacterial agents against resistant strains of bacteria [16, 17, 38]. For instance, numerous studies have shown that chalcones are more effective against resistant strains of bacteria than flavones or flavanones and that hydroxyl groups at the 2′ position are important for the antistaphylococcal activity of these compounds. In addition, neither fluorination nor chlorination at position 4′ of the chalcones B ring is reported to significantly affect antibacterial properties [39].

Evidence indicates that among the phenol derivatives analyzed in the* A. satureioides* extracts, achyrobichalcone could be responsible for the antibacterial activity. However, other structural analogues of this same class of flavonoids would need to be synthesized and examined before the effect of halogenation upon antibacterial activity can be properly assessed. For instance, methoxy groups reportedly drastically decrease the antibacterial activity of flavonoids [39], and these data may explain the lack of inhibition observed in 3-*O*-methyl-quercetin, the major phenolic compound in the freeze-dried extract.

Previous reports have suggested that the antibacterial properties of flavonoids such as quercetin may play a role in inhibiting nucleic acid synthesis [40]. Further, chalcones may exert antibacterial effects by changing the permeability of cellular membranes and damaging membrane function or inhibiting energy metabolism [41].

Although there are comparatively few studies regarding the mechanisms underlying flavonoid-induced antibacterial activity, numerous studies from the literature indicate that different natural products and phytochemicals (e.g., terpenes/terpenoids) may target different components and functions of the bacterial cell [42–44].

The intestinal microbiota are considered symbiotic in nature and are involved in various processes, including the breakdown and absorption of nutrients, production of vitamins and hormones, and prevention of colonization by pathogens. Failure to achieve or maintain this equilibrium between a host and its microbiota leads to dysbiosis, which has negative consequences for both intestinal and systemic health [45]. Our results show that the abundance and diversity of the gut microbiota in rats supplemented with the freeze-dried extract prepared from a hydroethanol extractive solution were not significantly different from those of the control. This is an important result because studies have shown that several diseases that are associated with altered barrier function and increased permeability of the epithelium are linked to changes in the microbiota population or reductions in the diversity of the microbiota [46]. The antioxidant activity of the extracts at low concentrations, ferrous ion-chelating ability, reduction equivalents for ferric ions to ferrous ions (via the FerroZine assay), and antimicrobial activities may contribute to the lack of differences in abundance and diversity observed for the gut microbiota. Most of the ingested dietary polyphenols are not absorbed in the small intestine and can interact with colonic microbiota, producing diverse metabolites with a variety of physiological roles [12] that can modulate gut microbiota [12, 13].

Fluorine, found in the* A. satureioides* extracts at a concentration of 17 mg/kg, has been used worldwide to prevent dental caries, attributable to the known in vitro inhibitory mechanisms against the production of bacterial acid [47]. Although further studies are needed, it appears that the extracts have anticarcinogenic effects depending on fluorine content.

In conclusion, we show evidence for the antimicrobial activity of* A. satureioides* freeze-dried extract obtained from a hydroethanol extractive solution against intestinal pathogens, which may be attributable, at least in part, to its antioxidant properties. Because these extracts do not produce any changes in the microbiota bacterial composition, future research is necessary regarding their potential use as coadjuvant agents for the treatment of intestinal diseases involving bacteria that present high rates of resistance to antibiotics.

## Figures and Tables

**Table 1:** Nutritional composition of *Achyrocline satureioides* inflorescences and the corresponding extracts (g/100** **g dry weight) ± standard deviation (SD).

Samples	Lipid (g/100 g)	Protein (g/100 g)	Reducing sugar (g/100 g)	Loss on drying (g/100 g)
In nature	11.0 ± 0.6^a^	1.74 ± 0.16^a^	17.27 ± 0.3^a^	10.9 ± 0.4^b^
Aqueous extract^*∗*^	5.8 ± 0.15^c^	0.97 ± 0.07^c^	17.22 ± 0.9^a^	17.0 ± 0.7^a^
Freeze-dried extract^*∗∗*^	10.8 ± 0.35^b^	1.3 ± 0.7^b^	16.6 ± 0.4^a^	17.8 ± 0.35^a^
Spray-dried extract^*∗∗*^	11.3 ± 0.2^a^	1.10 ± 0.2^c^	16.2 ± 0.6^a^	5.7 ± 0.2^c^

^*∗*^Aqueous extract: freeze-dried extract prepared from *A. satureioides* decoction. ^*∗∗*^Freeze-dried or spray-dried extracts prepared from *A. satureioides* extractive solution were obtained by macerating inflorescences in 80% ethanol. All extractive solutions were prepared using a plant : solvent ratio of 7.5% (w/v). Spray-dried extracts contained 50% extractives, 33.4% colloidal silicon dioxide, and 16.6% polysorbate 80; excipient percentages were considered in the analysis. Different letters in the same column indicate significant differences (*P* < 0.05) determined by Tukey's test of nutritional composition between extracts and the *A. satureioides* inflorescences.

**Table 2:** Total metal content in *Achyrocline satureioides* inflorescences and the corresponding extracts (kg^−1^ dry weight) ± standard deviation (SD).

Samples	Lead (mg kg^−1^)	Cadmium (mg kg^−1^)	Chrome (mg kg^−1^)	Fluoride (mg kg^−1^)
In nature	0.35 ± 0.04^b^	0.09 ± 0.01^a^	0.62 ± 0.05^b^	17.0 ± 1.8
Aqueous extract^*∗*^	0.38 ± 0.03^b^	0.10 ± 0.01^a^	0.45 ± 0.02^c^	17.0 ± 2.2
Freeze-dried extract^*∗∗*^	0.49 ± 0.06^a^	0.010 ± 0.001^b^	0.47 ± 0.01^c^	4.8 ± 0.8
Spray-dried extract^*∗∗*^	0.13 ± 0.02^c^	0.008 ± 0.001^b^	0.73 ± 0.06^a^	2.5 ± 0.3

^*∗*^Aqueous extract: freeze-dried extract prepared from *A. satureioides* decoction. ^*∗∗*^Freeze-dried or spray-dried extracts prepared from *A. satureioides* extractive solution were obtained by macerating inflorescences in 80% ethanol (v/v). All the extractive solutions were prepared with a plant : solvent ratio of 7.5% (w/v). Different letters in the same column indicate significant differences (*P* < 0.05) in the amount of metals between extracts and the *A. satureioides* inflorescences.

**Table 3:** Flavonoids and chalcone content in dried *Achyrocline satureioides* extracts (*μ*g/mg dry weight).

Samples	Quercetin	3-*O*-Methylquercetin	Luteolin	Achyrobichalcone	Total flavonoid + chalcone
Aqueous extract^*∗*^	15.68 ± 0.3^c^	27.05 ± 0.1^b^	5.5 ± 0.02^c^	6.0 ± 2.2^c^	54.23^c^
Freeze-dried extract^*∗∗*^	27.7 ± 0.6^b^	62.3 ± 0.5^a^	18.0 ± 0.01^b^	24.0 ± 0.8^a^	132.0^a^
Spray-dried extract^*∗∗*^	31.34 ± 0.02^a^	60.64 ± 0.7^a^	17.22 ± 0.06^a^	20.5 ± 0.3^b^	129.7^b^

^*∗*^Aqueous extract: freeze-dried extract prepared from *A. satureioides* decoction. ^*∗∗*^Freeze-dried or spray-dried extracts prepared from *A. satureioides* extractive solution were obtained by macerating inflorescences in ethanol 80% (v/v). Spray-dried extract contained 50% extractives, 33.4% colloidal silicon dioxide, and 16.6% polysorbate 80; excipient percentages were considered in the analysis. All the extractive solutions were prepared with a plant : solvent ratio of 7.5% (w/v). Different letters in the same column indicate significant differences (*P* < 0.05) in the amount of flavonoids between extracts.

**Table 4:** Antibacterial effect of freeze-dried extracts prepared from an *Achyrocline satureioides* extractive solution obtained by macerating inflorescences in 80% ethanol (v/v). Antibiotics (chloramphenicol, amoxicillin, and ciprofloxacin), as well as 3-O-methylquercetin (primary flavonoid in the extracts), were tested against strains from gram-positive and gram-negative bacteria. Antibacterial activities were classified as “no activity” (−), “modest” (+), “clear” (++), or “strong” (+++), corresponding to inhibition zones of ≤1, 2–4, 5–10, and >10 mm, respectively.

Bacteria and strains	Flavonoid extracts and antibiotics				
	*Achyrocline satureioides* [200 mg/mL]	3-O-methylquercetin	Amoxicillin	Chloramphenicol	Ciprofloxacin
Gram-positive					
*Bacillus cereus* ATCC 9634	+	−	−	+++	+++
*Staphylococcus aureus* ATCC 1901	+	−	−	+++	+++
Gram-negative					
*Salmonella enteritidis* ATCC 13 076	+	−	++	+++	+++
